# Correction: Blood-brain barrier breakdown in non-enhancing multiple sclerosis lesions detected by 7-Tesla MP2RAGE ΔT_1_ mapping

**DOI:** 10.1371/journal.pone.0264452

**Published:** 2022-02-22

**Authors:** Seongjin Choi, Margaret Spini, Jun Hua, Daniel M. Harrison

## Notice of Republication

This article was republished on February 10, 2022, to correct errors throughout the article that were introduced during the revision process. The authors apologize for the errors. Please download this article again to view the correct version. The originally published, uncorrected article and the republished, corrected articles are provided here for reference.

## Supporting information

S1 File Originally published, uncorrected article. (PDF)

S2 File Republished, corrected article. (PDF)
