# Supplementary material for: Blood-brain barrier breakdown in non-enhancing multiple sclerosis lesions detected by 7-Tesla MP2RAGE ΔT1 mapping
Source: PLoS One. 2021 Apr 26;16(4):e0249973. doi: 10.1371/journal.pone.0249973 (PMC8075220; doi:10.1371/journal.pone.0249973)
Supplement: S1 Table — (DOCX) [file pone.0249973.s001.docx]

**S1 Table. Partial correlation of ΔT_1_ with WML volume and count.**

| Tissue | T_1_ metric | WML volume | | | WML count | | | WML volume/count | | |
| --- | --- | --- | --- | --- | --- | --- | --- | --- | --- | --- |
|  | (per subject) | partial r | p | adjusted p | partial r | p | adjusted p | partial r | p | adjusted p |
| WML | mean ∆T_1_ | 0.147 | 0.341 | 0.432 | -0.206 | 0.180 | 0.373 | 0.233 | 0.128 | 0.373 |
|  | variance ∆T_1_ | 0.199 | 0.196 | 0.373 | **0.601** | **< 0.001** | **< 0.001** | -0.156 | 0.311 | 0.432 |
|  | median ∆T_1_ | 0.137 | 0.374 | 0.432 | -0.282 | 0.063 | 0.317 | 0.254 | 0.097 | 0.362 |
|  | IQR ∆T_1_ | 0.197 | 0.199 | 0.373 | **0.496** | **< 0.001** | **0.005** | -0.137 | 0.374 | 0.432 |
|  | kurtosis ∆T_1_ | 0.185 | 0.229 | 0.381 | 0.017 | 0.912 | 0.912 | 0.119 | 0.441 | 0.472 |
| NEL | mean ∆T_1_ | 0.190 | 0.217 | 0.365 | -0.192 | 0.212 | 0.365 | 0.259 | 0.090 | 0.274 |
|  | variance ∆T_1_ | 0.122 | 0.428 | 0.494 | **0.589** | **< 0.001** | **< 0.001** | -0.210 | 0.170 | 0.365 |
|  | median ∆T_1_ | 0.145 | 0.346 | 0.490 | -0.279 | 0.067 | 0.274 | 0.258 | 0.091 | 0.274 |
|  | IQR ∆T_1_ | 0.189 | 0.219 | 0.365 | **0.492** | **< 0.001** | **0.005** | -0.142 | 0.359 | 0.490 |
|  | kurtosis ∆T_1_ | -0.025 | 0.871 | 0.871 | -0.128 | 0.407 | 0.494 | 0.038 | 0.807 | 0.865 |
| NAWM | mean ∆T_1_ | 0.146 | 0.344 | 0.567 | 0.208 | 0.175 | 0.567 | -0.022 | 0.887 | 0.887 |
|  | variance ∆T_1_ | 0.179 | 0.245 | 0.567 | **0.350** | **0.020** | 0.296 | -0.088 | 0.570 | 0.777 |
|  | median ∆T_1_ | 0.195 | 0.204 | 0.567 | 0.239 | 0.119 | 0.567 | -0.023 | 0.884 | 0.887 |
|  | IQR ∆T_1_ | 0.139 | 0.367 | 0.567 | 0.232 | 0.129 | 0.567 | -0.040 | 0.796 | 0.887 |
|  | kurtosis ∆T_1_ | -0.151 | 0.327 | 0.567 | -0.136 | 0.378 | 0.567 | -0.066 | 0.672 | 0.839 |
| cGM | mean ∆T_1_ | **0.394** | **0.008** | 0.061 | **0.351** | **0.019** | 0.073 | 0.138 | 0.372 | 0.445 |
|  | variance ∆T_1_ | 0.219 | 0.153 | 0.299 | **0.420** | **0.005** | 0.061 | -0.134 | 0.386 | 0.445 |
|  | median ∆T_1_ | **0.324** | **0.032** | 0.097 | 0.234 | 0.126 | 0.299 | 0.151 | 0.329 | 0.445 |
|  | IQR ∆T_1_ | 0.216 | 0.160 | 0.299 | **0.353** | **0.019** | 0.073 | -0.099 | 0.521 | 0.558 |
|  | kurtosis ∆T_1_ | -0.158 | 0.305 | 0.445 | -0.161 | 0.295 | 0.445 | 0.076 | 0.623 | 0.623 |
| WML = white matter lesion; NEL = non-enhancing lesion; NAWM = normal-appearing white matter; cGM = cortical gray matter. partial r = Pearson's correlation coefficient after controlling for age, sex, and symptom duration; p = p-value; adjusted p = p-value corrected for multiple comparison (false discovery rate: FDR) | | | | | | | | | | |
| All p-values are given with actual numbers except values < 0.001. Coefficients with p-values less than 0.05 are shown in bold face. | | | | | | | | | | |
