# Supplementary material for: Blood-brain barrier breakdown in non-enhancing multiple sclerosis lesions detected by 7-Tesla MP2RAGE ΔT1 mapping
Source: PLoS One. 2021 Apr 26;16(4):e0249973. doi: 10.1371/journal.pone.0249973 (PMC8075220; doi:10.1371/journal.pone.0249973)
Supplement: S2 Table — (DOCX) [file pone.0249973.s002.docx]

**S2 Table. Partial correlation of per subject ΔT_1_ with clinical measures.**

| Tissue | T_1_ metric | EDSS | | | SDMT | | | PASAT | | | MFIS | | |
| --- | --- | --- | --- | --- | --- | --- | --- | --- | --- | --- | --- | --- | --- |
|  | (per subject) | partial rho | p | adjusted p | partial r | p | adjusted p | partial r | p | adjusted p | partial r | p | adjusted p |
| WML | mean ∆T_1_ | -0.234 | 0.127 | 0.403 | 0.003 | 0.985 | 0.985 | 0.169 | 0.279 | 0.568 | -0.161 | 0.302 | 0.568 |
|  | variance ∆T_1_ | **0.319** | **0.035** | 0.243 | -0.092 | 0.554 | 0.776 | -0.006 | 0.968 | 0.985 | 0.256 | 0.097 | 0.378 |
|  | median ∆T_1_ | **-0.307** | **0.043** | 0.250 | 0.043 | 0.781 | 0.855 | 0.199 | 0.202 | 0.504 | -0.096 | 0.541 | 0.776 |
|  | IQR ∆T_1_ | **0.335** | **0.026** | 0.229 | -0.213 | 0.164 | 0.479 | -0.065 | 0.677 | 0.855 | 0.183 | 0.240 | 0.560 |
|  | kurtosis ∆T_1_ | **-0.358** | **0.017** | 0.199 | 0.274 | 0.072 | 0.313 | 0.049 | 0.757 | 0.855 | 0.029 | 0.854 | 0.906 |
| NEL | mean ∆T_1_ | -0.187 | 0.223 | 0.507 | 0.012 | 0.936 | 0.936 | 0.178 | 0.253 | 0.507 | -0.115 | 0.463 | 0.705 |
|  | variance ∆T_1_ | 0.242 | 0.114 | 0.414 | -0.114 | 0.460 | 0.705 | -0.030 | 0.848 | 0.873 | 0.175 | 0.261 | 0.507 |
|  | median ∆T_1_ | -0.297 | 0.050 | 0.254 | 0.044 | 0.776 | 0.849 | 0.199 | 0.200 | 0.500 | -0.088 | 0.573 | 0.771 |
|  | IQR ∆T_1_ | **0.327** | **0.030** | 0.213 | -0.214 | 0.163 | 0.438 | -0.067 | 0.671 | 0.804 | 0.176 | 0.260 | 0.507 |
|  | kurtosis ∆T_1_ | **-0.382** | **0.011** | 0.118 | **0.444** | **0.003** | 0.089 | 0.035 | 0.822 | 0.872 | -0.251 | 0.105 | 0.414 |
| NAWM | mean ∆T_1_ | 0.246 | 0.107 | 0.466 | 0.108 | 0.485 | 0.798 | 0.113 | 0.470 | 0.798 | 0.057 | 0.717 | 0.809 |
|  | variance ∆T_1_ | **0.312** | **0.039** | 0.276 | -0.091 | 0.559 | 0.798 | -0.122 | 0.435 | 0.798 | 0.153 | 0.327 | 0.798 |
|  | median ∆T_1_ | 0.203 | 0.185 | 0.589 | 0.105 | 0.499 | 0.798 | 0.124 | 0.429 | 0.798 | 0.089 | 0.570 | 0.798 |
|  | IQR ∆T_1_ | **0.326** | **0.031** | 0.268 | -0.123 | 0.427 | 0.798 | -0.146 | 0.350 | 0.798 | 0.180 | 0.248 | 0.699 |
|  | kurtosis ∆T_1_ | **-0.333** | **0.027** | 0.268 | 0.102 | 0.509 | 0.798 | 0.176 | 0.259 | 0.699 | -0.300 | 0.051 | 0.297 |
| cGM | mean ∆T_1_ | 0.132 | 0.393 | 0.603 | 0.050 | 0.745 | 0.790 | 0.191 | 0.220 | 0.506 | 0.078 | 0.619 | 0.722 |
|  | variance ∆T_1_ | 0.268 | 0.078 | 0.356 | -0.090 | 0.563 | 0.694 | -0.172 | 0.270 | 0.525 | 0.128 | 0.413 | 0.603 |
|  | median ∆T_1_ | 0.019 | 0.900 | 0.927 | 0.087 | 0.575 | 0.694 | 0.215 | 0.166 | 0.506 | 0.009 | 0.955 | 0.955 |
|  | IQR ∆T_1_ | **0.306** | **0.043** | 0.356 | -0.152 | 0.325 | 0.599 | -0.186 | 0.231 | 0.506 | 0.208 | 0.181 | 0.506 |
|  | kurtosis ∆T_1_ | -0.279 | 0.067 | 0.356 | 0.207 | 0.177 | 0.506 | 0.203 | 0.192 | 0.506 | -0.180 | 0.249 | 0.512 |

| Tissue | T_1_ metric | 9HPTDOM | | | 9HPTNONDOM | | | 25FTW | | |
| --- | --- | --- | --- | --- | --- | --- | --- | --- | --- | --- |
|  | (per subject) | partial r | p | adjusted p | partial r | p | adjusted p | partial r | p | adjusted p |
| WML | mean ∆T_1_ | -0.157 | 0.308 | 0.568 | -0.057 | 0.712 | 0.855 | -0.056 | 0.719 | 0.855 |
|  | variance ∆T_1_ | **0.367** | **0.014** | 0.199 | 0.045 | 0.773 | 0.855 | 0.205 | 0.182 | 0.490 |
|  | median ∆T_1_ | -0.239 | 0.118 | 0.403 | -0.072 | 0.643 | 0.855 | -0.144 | 0.350 | 0.612 |
|  | IQR ∆T_1_ | **0.371** | **0.013** | 0.199 | 0.102 | 0.509 | 0.776 | 0.296 | 0.051 | 0.255 |
|  | kurtosis ∆T_1_ | -0.118 | 0.445 | 0.741 | -0.094 | 0.543 | 0.776 | -0.166 | 0.280 | 0.568 |
| NEL | mean ∆T_1_ | -0.160 | 0.298 | 0.527 | -0.064 | 0.682 | 0.804 | -0.062 | 0.689 | 0.804 |
|  | variance ∆T_1_ | **0.377** | **0.012** | 0.118 | 0.057 | 0.715 | 0.808 | 0.217 | 0.156 | 0.438 |
|  | median ∆T_1_ | -0.239 | 0.118 | 0.414 | -0.073 | 0.638 | 0.804 | -0.145 | 0.347 | 0.578 |
|  | IQR ∆T_1_ | 0.370 | **0.013** | 0.118 | 0.103 | 0.506 | 0.737 | 0.296 | 0.051 | 0.254 |
|  | kurtosis ∆T_1_ | -0.159 | 0.301 | 0.527 | -0.097 | 0.530 | 0.742 | -0.226 | 0.141 | 0.438 |
| NAWM | mean ∆T_1_ | 0.079 | 0.610 | 0.809 | -0.011 | 0.943 | 0.958 | 0.061 | 0.696 | 0.809 |
|  | variance ∆T_1_ | **0.484** | **0.001** | **0.031** | 0.047 | 0.762 | 0.809 | 0.224 | 0.143 | 0.501 |
|  | median ∆T_1_ | 0.047 | 0.761 | 0.809 | -0.008 | 0.958 | 0.958 | 0.052 | 0.736 | 0.809 |
|  | IQR ∆T_1_ | 0.339 | 0.024 | 0.268 | 0.048 | 0.759 | 0.809 | 0.276 | 0.069 | 0.346 |
|  | kurtosis ∆T_1_ | -0.098 | 0.525 | 0.798 | -0.058 | 0.709 | 0.809 | -0.238 | 0.120 | 0.466 |
| cGM | mean ∆T_1_ | 0.068 | 0.661 | 0.746 | -0.186 | 0.227 | 0.506 | -0.053 | 0.733 | 0.790 |
|  | variance ∆T_1_ | **0.477** | **0.001** | **0.037** | 0.119 | 0.441 | 0.603 | 0.244 | 0.111 | 0.430 |
|  | median ∆T_1_ | -0.117 | 0.448 | 0.603 | -0.127 | 0.412 | 0.603 | -0.120 | 0.439 | 0.603 |
|  | IQR ∆T_1_ | **0.435** | **0.003** | 0.056 | 0.110 | 0.477 | 0.619 | **0.308** | **0.042** | 0.356 |
|  | kurtosis ∆T_1_ | -0.266 | 0.081 | 0.356 | -0.136 | 0.379 | 0.603 | -0.286 | 0.060 | 0.356 |

WML = white matter lesion; NEL = non-enhancing lesion; NAWM = normal-appearing white matter; cGM = cortical gray matter; IQR = inter-quartile range; partial rho = Spearman's rho coefficient after controlling for age, sex, and symptom duration; partial r = Pearson's correlation coefficient after controlling for age, sex, and symptom duration; p = p-values; adjusted p = p-value corrected for multiple comparison (false discovery rate: FDR); EDSS = Expanded Disability Status Scale; SDMT = Symbol Digit Modalities Test; PASAT = Paced Auditory Serial Addition Test; MFIS = Modified Fatigue Impact Scale; 9HPTDOM = 9-hole peg test for dominant hand; 9HPTNONDOM = 9-hole peg test for non-dominant hand; 25FTW = timed 25-foot walk. All p-values are given with actual numbers except values < 0.001. Coefficients with p-values less than 0.05 are shown in bold face.
