# Supplementary material for: Blood-brain barrier breakdown in non-enhancing multiple sclerosis lesions detected by 7-Tesla MP2RAGE ΔT1 mapping
Source: PLoS One. 2021 Apr 26;16(4):e0249973. doi: 10.1371/journal.pone.0249973 (PMC8075220; doi:10.1371/journal.pone.0249973)
Supplement: Attachment — Submitted filename: Response to Reviewers.docx [file pone.0249973.s003.docx]

Dear Editorial Staff and Reviewers,

We thank you for your careful review of our submission. We have worked to make the suggested changes, and feel that the paper is much improved due to your suggestions. We hope you agree. Our responses to your comments are shown below your comment in italics. Subsequent changes in the manuscript document are highlighted in yellow for your convenience.

Editor:

In addition to the comments raised by the Reviewers, please also address the following comments:

1. The word “gender” is used throughout the manuscript. Usually, “sex” (the biological designation) is meant. “Gender” is the social construct and is rarely relevant in neurologic disease. Please revise the text to use “sex” rather than “gender” throughout.

*This has been fixed.*

2. The "Symbol Digit Modalities Test" is incorrectly referred to as the "symbol digits modalities test" (note the lack of pluralization of digits and capitalization in the correct spelling).

*This has been fixed.*

3. The point about potentially inhibiting hypothesis-generating research due to multiple comparisons is understood, some correction is warranted in the context of your manuscript. Table 3 and Table 4 have 45 and 105 tests, respectively. While Bonferroni correction would indeed be overly conservative, something like false discovery rate correction be considered.

*We understand your concern, and as such, have performed multiple comparison adjustments. We still do have strong concerns that such an approach in a smaller cohort study has the potential to overcorrect and result in lack of future research into an area that might have merit. As such, we have provided both raw and adjusted p values, and hope that readers and reviewers alike can come to their own conclusions.*

4. P-values, by definition, cannot be equal to 0.0, as reported in Table 4. Please update accordingly.

*We apologize for this error. Those p values fell below the number of significant digits shown in the table, and should have been represented as < a certain value. This has been corrected.*

5. While the comments about the benefit of the MP2RAGE sequence with respect to not requiring co-registration for T1 measurements is acknowledged, this point is somewhat weakened later on when you refer to the subtraction of T1 maps in the context of the current manuscript, which does require co-registration. Consider mentioning this.

*We apologize if this phrasing was unclear. Of course, any technique measuring the change in T1 with contrast administration will need to compare a pre-contrast image with a post-contrast image, and those will have to be co-registered. The point being made in the introduction was that other techniques, such as SPGR, require multiple acquisitions that need to be co-registered just to get a T1 map. Thus, one must have multiple acquisitions pre-contrast, co-register and process those to get a pre-contrast T1 map, and then repeat the process for a post-contrast T1 map, and then do a 3^rd^ co-registration step to register pre- and post-contrast T1 maps together to calculate a difference map. MP2RAGE produces an inherent T1 map and does not require additional registration to do so. So, the one and only co-registration that is needed is the comparison between pre- and post-contrast. We have added some additional wording to the comment about SPGR in the introduction to make this clearer.*

Reviewer #1: Dr Choi and colleagues report a cross-sectional study to investigate if there is a BBB disruption in non-enhancing lesions (NEL) evaluating changes in T1 relaxation time in pre-and post-contrast MRI using MP2RAGE at 7T. They found that subtraction of pre-and post-contrast T1 maps, ΔT1, was greater in NEL than in NAWM and correlated with EDSS. While it is a novel approach, there are former articles at lower resolution MRI showing that NELs have increased permeability compared to NAWM.

I have major concerns.

• Abstract: The authors propose that NEL ΔT1 should be further evaluated as a biomarker for disease severity and treatment effect in MS. I would rather say that it should be evaluated as a possible biomarker of persistently disrupted BBB.

*Thank you for the suggested change. We have rephrased.*

• Introduction: They state: -The existence of chronic-active lesions, along with the relative inability to differentiate these from inactive lesions on standard MRI sequences, demonstrates need additional in vivo markers for chronic inflammation in MS-. However, there are articles that showed in vivo and postmorten paramagnetic rims in susceptibility weighted imaging at 7, that correlated with chronic active lesions in pathological analysis. The presence of paramagnetic RIMS was also assessed at 3T MRI. A paragraph describing these results and data on slowly expanding lesions should be added.

*We agree with the reviewer that susceptibility imaging visualization of lesions with paramagnetic rims is helpful in visualizing chronic-active lesions (although this finding is also reported in active lesions) and that our mention of chronic-active lesions in the introduction without discussing the imaging finding was insufficient. As opposed to your suggestion, however, since our study is aimed at quantification of BBB quantification rather than binary classification of chronic-active vs inactive lesions, we elected to remove this discussion in the introduction and replace with a more relevant discussion of BBB breakdown and gadolinium.*

• Materials and methods: Was MP2RAGE the only sequence acquired?

*The images acquired in this study were acquired as part of a multi-modal imaging study. Comprehensive analysis of all image modalities acquired would be beyond the scope of this paper. However, we do agree that we should have mentioned information about the MPFLAIR sequence acquired, as this was used in tandem with T1 for lesion identification. This was updated.*

• Results: Data on the disease duration should be included. What percentage of progressive patients where under treatment? These data are important for the interpretation of the results.

*We thank you for the suggestion. Table 1 now includes disease duration (as symptom duration) and Tables 3 and 4 include raw and adjusted correlations (adjusted for age, sex, and disease duration). The ‘Results’ section, now, includes the percentage information of patients on treatment (RRMS vs. PMS).*

• Discussion:
I. Paragraph-4 Authors claim that the magnitude and variability of ΔT1 is associated with the severity of MS-related disability, MS-phenotype and the use of immunomodulatory MS treatment. To better analyze the results, it is important to include disease duration and percentage of progressive MS under treatment.

*We thank you for the suggestions. Table 4 now includes the partial correlations to account for age, sex, and disease duration. We also have now broken down the percentage of subjects who were on treatment in the relapsing and progressive subgroups separately in the new version of Table 1.*

II. Paragraph-5 As they found that ΔT1 revealed less BBB breakdown as a function of immunomodulatory therapy, they highlight the potential applicability of this technique as an outcome measure in clinical trials, and in clinical practice. The authors should deeply clarify that MP2RAGE at 7T is not currently feasible in clinical trials setting or in clinical practice. As they mention in the last sentence of that paragraph, their findings need to be replicated first at 3T.

*Thank you for the comment. We hope that the last half of paragraph 5 builds towards this point. However, we can see that this point needs to be made stronger. As such, we have added one additional sentence at the end of the paragraph.*

III. Paragraph-7 They propose that changes in ΔT1 may be due to increased cytokine release and microglial activity due to chronic inflammation, or more permanent disruptive changes to the BBB that occurred at lesion formation. They claim that the differentiation between these two processes would likely require in vivo imaging followed by post-mortem histopathology. However, I think that a good approach to elucidate possible mechanisms involved would be to see if there is a gradient of ΔT1 in individual lesions, if a greater ΔT1 is detected at the edge of the lesion, this would be in favor of chronic inflammation in the border of the lesion. Furthermore, if susceptibility-weighted images show paramagnetic rims in NEL with greater ΔT1, it would suggest chronic inflammation in the edge of the lesion. Could the authors do a more detailed analysis?

*We thank you for the suggestion. Unfortunately, we suggest that such an analysis would be well beyond the scope of this paper and also may not provide any definitive conclusion. Regarding scope, as GRE images were not reviewed for paramagnetic rims and paramagnetic rim lesions were not separately masked, this would add multiple additional processing/identification/masking/registration/analysis steps – all of which would have to be performed, described, and evaluated in detail on their own in this already large paper (both in size and concept). As a first description of the ΔT1 concept requires much more detail on MP2RAGE ΔT1 methods and analysis, we feel there is not sufficient room to do so at this time. We do agree that, perhaps, in a future analysis, once the ΔT1 concept is described in this paper and this paper can be referred to, a multi-modal analysis of the type suggested could be done. Further, it should be noted that while lesions with paramagnetic rims do indeed correlate well with chronic-active lesions at autopsy, the finding of paramagnetic rims also has been described in acutely inflamed lesions, so specificity may complicate interpretation.*

*Regarding the spatial analysis suggested – unfortunately that is not possible with the current WML masks created for this paper. As described in the methods, each individual WML was not mapped on its own. Rather, a full comprehensive WML mask was created for each individual subject. The full 3D volume of this mask and an enhancing WML mask for each subject were subtracted to create the full 3D volume for NELs for each subject. The analysis suggested would require each individual lesion in each individual subject to separated into individual volumes and then creation of a geometric methodology for separating the inner and outer portions of those volumes. While that could be the goal of a later undertaking and report, the idea for which we thank the reviewer, this would not be possible at this time with this dataset. We should note that our original Discussion section (last paragraph) acknowledges the limitation of the full volume approach, rather than a lesion-by-lesion analysis, but also discusses the biases/limitations of lesion-by-lesion analyses.*

*Although this does not entirely answer the reviewer’s request, though, it should be noted that a qualitative review of ΔT1 patterning was performed and is described in the methods and results, and a clear inner vs outer pattern was not clearly seen (examples in Figure 2). However, a new, comprehensive, lesion-by-lesion quantitative volumetric study could and should be done in the future, which will answer your question.*

• Conclusion: I would focus the conclusion on that using MP2RAGE at 7T, persistent disruption of the BBB was found. The last sentence mentions that there are currently no stablished biomarkers, I would remove it, there are promising biomarkers.

*Thank you for the suggestion. We have reworded this section.*

Reviewer #2: Thank you for this interesting study.
Please reformat Table 1 to show the variables subdivided by groups of MS category. It is important to know the relative ages and disability levels

*Thank you for the suggestion. This change has been made.*

The average age of this cohort was 46.2 (11.2). WM lesions of vascular origin may have also occurred in this cohort. Did you make an attempt to distinguish these from MS lesions?

*Thank you for pointing this out. As there is no validated method to provide an absolute distinction between vascular lesions and MS lesions, this was not performed. One could fully restrict the analysis ONLY to periventricular and juxtacortical WMLs and eliminate all subcortical WMLs to reduce the chance of overlap. However, this would eliminate a large volume of WMLs, does not have 100% specificity, and, since the categorization of lesions as such would be manual, would introduce reviewer biases to the analysis. As such, we elect to remain with a more agnostic, comprehensive approach, including all WMLs. The Central Vein Sign has also been proposed as a means for differentiation of vascular WMLs from demyelinating lesions. However, while this finding is seen in a higher proportion of demyelinating lesions than vascular lesions, it is not a ubiquitous finding in demyelinating lesions, and, as such, could not fully differentiate each and every lesion for the type of distinction the reviewer suggests.

Given such limitations, we have preferred to acknowledge your comment as a limitation of this (and any similar WML) analysis at the end of the discussion section. This has been added.*

Why did you chose EDSS = 5.0 as a threshold for binarization? This is not particularly clinically meaningful. Since 3.0 was the median in your cohort (and 6.5 the max), and since this is more meaningful (mild vs moderate disability), this might be a more appropriate threshold.

*We appreciate reviewer’s great suggestion. We regrouped the disability groups reflecting reviewer’s suggestion (median EDSS = 3) and we observed more significant difference between the new two groups. We reworked the part of the Table 5 and corresponding section of the manuscript reflecting the results changed by introducing new threshold.*

The phrase ‘inter-subject mean of per subject median’ is not well explained. Suggest rephrase or explain more fully in methods.

*We understand how this can be confusing. We rephrased it in the note on the Table 2: “All measures in the second column indicate mean and median values of 47 individual median T1 values collected for each tissue.” We hope this clarifies.*

You found a pre/post-contrast difference in NAWM when using the median but not the mean – how do you explain this?

*To help answer this query, the distribution of 47 individual intra-subject pre-/post contrast median T_1_ values within NAWM are shown in the following figure. When comparing the inter-subject pre-contrast and post-contrast mean T_1_ value by t-test, a difference in means was not found. When comparing the inter-subject pre-contrast and post-contrast median T1 value by Wilcoxon test, a difference in medians was found. As one can see from the distribution, the peak of the histograms (median) can easily be visually separated. The pre-contrast peak has a wider, almost bimodal spread, whereas the post-contrast peak is sharper and more unimodal. Thus, the mean and median value for post-contrast are similar, whereas the mean value for pre-contrast would be ‘shifted to the left’ compared to its median, making it more similar to the post-contrast mean value. The differences between the two statistical tests are due to which central tendency they measure and how distributions are handled by parametric and non-parametric tests.*

You report that delta T1 was significantly greater in NELs than in NAWM. But NELs also had a higher pre-contrast T1 than NAWM (as expected). Therefore, if both NELs and NAWM had the same permeability, and accumulated the same concentration of contrast, NELs would have a higher delta T1 simply because of the higher pre-contrast T1 (about double – though you find a bigger change than this). Presumably in controls you find a higher delta T1 in GM vs WM? You should report delta T1 for NAGM in this cohort and compare to NAWM. I am concerned that using delta T1 to make inferences about BBB permeability is difficult. You report a weak correlation between pre-contrast T1 and delta T1 – this deserves more discussion and a figure. Advise redo the correlation but within tissue types – i.e. within NAWM, GM, etc. If the correlation is weak in NELs, then this may be due to heterogeneity in some other factor – like permeability (or vascular surface area). This would fit with your other findings of lesional heterogeneity.

*We thank the reviewer for the comment. As suggested, we have now added an analysis of cortical GM into this paper for comparison throughout. As expected, and as reported in other analyses of T1 shifts with gadolinium in the past, ΔT1 is seen in cortical GM. The T1 shift seen in cortical GM has previously been attributed to vascularity, cortical GM has higher vascular perfusion compared to WM.*

*Regarding the discussion about ΔT1 being potentially driven primarily by the pre-contrast T1 status of the tissue (and WMLs being higher), we previously intended our discussion of Figure 3 to help resolve this concern. We have made revisions to Figure 3 per your suggestions and discuss this more robustly in the new version of the paper. As one can see from this figure, there is only a weak correlation between pre-contrast T1 in NELs and the ΔT1 seen in that tissue. If the ΔT1 was mostly just an effect of the pre-contrast T1 value, this correlation should have been far stronger. As suggested by the reviewer, we have now done the same analysis for NAWM and cortical GM. These tissues also showed a weak correlation, however, the strength of correlation of pre-contrast T1 and ΔT1 in these tissues was greater than for NELs, suggesting that NEL ΔT1 is less dependent on this effect than NAWM and cortical GM and needs an additional explanation. We propose this is heterogeneity in BBB breakdown.*

*Please see the revised Figure 3 and additional language in the results and discussion.*

What was the rationale for a 10 minute gap between injection and post-contrast scan? Would a longer gap have influenced results?

*We would refer the reviewer to paragraphs 3 and 4 of the Discussion section, in which discussion of the timing of acquisition after contrast was discussed, including the reason for the 10 minute gap. In addition to the tissue permeability reason discussed there, this was also due to practical considerations, and the need to obtain additional sequences before the MP2RAGE scan for clinical patient care. As discussed in paragraph 4 of the Discussion, timing of contrast can influence results, and previous studies showed a lack of significant T1 shortening at earlier time points (i.e. 5 min). Given the mean distribution half-life of gadolinium, sequences acquired with a longer gap than 10 minutes would likely have influence results, also, but we cannot know for sure without having acquired such sequences. We have added an additional comment to this paragraph in the Discussion to acknowledge this limitation.*

I do not see that you acquired a T2-weighted or FLAIR sequence for lesion detection. If lesion masks were created using T1 information only, it is likely that some lesions were misclassified as NAWM, and that the lesions mask was biased towards black holes. You report the median T1 of lesions to be 2430 ms – this is very long and suggests that a significant number of black holes were present. The leakage behaviour of black holes is unlikely to be representative of T1-isointense lesions.

*Thank you for this comment. In an oversight, we neglected to include a description of acquisition and use of MPFLAIR sequences alongside the MP2RAGE sequences. While masks were drawn on T1 images, MPFLAIR was available alongside for guidance/assistance. While the reviewer would be correct if a TSE T1-weighted image was used, which would only identify ‘black hole’ type lesions, gradient-echo-based T1 sequences (like MPRAGE and MP2RAGE) should result in visualization of all demyelinated lesions and not be biased in this manner [consider Sheena L Dupuy et al. Eur J Radiol. 2015 Aug.]. We have added a bit more language to the methods to clarify the process. We also have cited our recent peer reviewed study (Spini et al. Journal of Neurology 2020), in which we reported that 7T MP2RAGE T1 lesion masks are viable alternative to FLAIR lesion masks. In this paper, all lesions were identified on both sequences. FLAIR lesion volumes were slightly greater, mostly due to artifacts in the brainstem and temporal lobes. Despite this, FLAIR and T1 lesion masks were highly correlated and equally related to disability.*

Has the raw data been provided? I cannot see in this manuscript. I assume this will follow in a supplementary material.

*All raw MRI data including pre-, post-, and delta T1 metrics have been prepared in a spreadsheet provided to the journal.*
