# Supplementary material for: Blood-brain barrier breakdown in non-enhancing multiple sclerosis lesions detected by 7-Tesla MP2RAGE ΔT1 mapping
Source: PLoS One. 2021 Apr 26;16(4):e0249973. doi: 10.1371/journal.pone.0249973 (PMC8075220; doi:10.1371/journal.pone.0249973)
Supplement: Attachment — Submitted filename: Response to Reviewers_DeltaT1-3-25-21.docx [file pone.0249973.s004.docx]

Dear Editorial Staff and Reviewers,

We thank you for your careful review of our submission and the recent positive review. We have made the suggested changes and have submitted both a “track changes” version of the document and a “clean version of the document for your final review. Your query is below and our response is in *italic* script.

Review Comments to the Author

Please use the space provided to explain your answers to the questions above. You may also include additional comments for the author, including concerns about dual publication, research ethics, or publication ethics. (Please upload your review as an attachment if it exceeds 20,000 characters)

Reviewer #1: 
I appreciate the detailed response.
I accept that this analysis is beyond the scope of this paper, however, it would be helpful if you could add a brief comment on how susceptibility-weighted imaging (presence of paramagnetic RIM) could help elucidate whether changes in ΔT1 in NEL may be due to chronic disrupted BBB or due to chronic inflammation.
As you mention, enhancing lesions may show a transient paramagnetic RIM, however, here, you want to discriminate the possible underlying processes in the ΔT1 changes seen in NEL.

The authors have addressed my other comments.

*We thank you for the thoughtful suggestion. We have added additional language to two of the paragraphs of the Discussion section in which chronic BBB disruption vs chronic inflammation were being discussed to address this point and propose future research that may tease this out. We hope this language is found to be acceptable.*

Reviewer #2: The authors have addressed all the comments clearly and I recommend that the manuscript be accepted.

*Thank you for your careful review and approval.*
